# Clinical efficacy of manual reverse closed reduction and robot-assisted cannulated screw fixation for valgus-impacted osteoporotic femoral neck fractures in the elderly: a retrospective cohort study

**DOI:** 10.3389/fsurg.2026.1830958

**Published:** 2026-07-10

**Authors:** Yuxiang Kang, Jin Qian, Kang Xu, Zhipeng Ren, Qiang Dong, Yinguang Zhang

**Affiliations:** 1 Department of Sports Injury and Arthroscopy, Tianjin Hospital, Tianjin, China; 2 Department of Orthopaedics, Tianjin Hospital, Tianjin, China

**Keywords:** cannulated screw fixation, femoral neck fractures, femoral neck shortening, reverse closed reduction, robot-assisted internal fixation, valgus-impacted fractures

## Abstract

Achieving anatomical restoration in valgus-impacted femoral neck fractures remains a clinical challenge, particularly in the elderly. This retrospective study evaluated a “dual-optimization” strategy—integrating manual reverse closed reduction with robotic-assisted internal fixation—compared to conventional *in-situ* freehand fixation. Seventy-two patients were divided into the reduction-assisted (RA, *n* = 36) and *in-situ* freehand (IF, *n* = 36) groups. While the RA group required longer operative times (1.74 ± 0.63 h vs. 1.29 ± 0.37 h, *p* < 0.05), it demonstrated superior technical precision with significantly fewer fluoroscopy sessions (33.28 ± 7.71 vs. 56.56 ± 19.71) and guide-pin adjustments (0.17 ± 0.38 vs. 1.89 ± 1.09, *p* < 0.0001). Radiologically, the RA group achieved effective deformity correction (valgus and posterior tilt angles, *p* < 0.0001) and exhibited higher screw placement accuracy (5.92 ± 3.75° vs. 11.14 ± 5.28°) and superior screw dispersion (22.10 ± 1.47% vs. 18.83 ± 1.22%, *p* < 0.05). At 24–36 months follow-up, the RA group showed significantly less femoral neck shortening (2.5 ± 2.1 mm vs. 9.1 ± 3.8 mm, *p* < 0.0001) and higher Harris Hip Scores (82.00 ± 11.44 vs. 73.61 ± 15.07, *p* < 0.05). Furthermore, the RA group had markedly lower incidences of femoral head necrosis (5.56% vs. 25.00%), non-union (8.33% vs. 27.78%), and fixation failure (5.56% vs. 22.22%, *p* < 0.05). In conclusion, within the limitations of this cohort, combining manual reverse reduction with robotic assistance synergistically may provide clinical benefits by improving surgical precision and maintaining femoral neck anatomy, showing a positive tendency toward better functional recovery and reduced complications for geriatric valgus-impacted fractures.

## Introduction

Hip fractures represent one of the most debilitating osteoporotic injuries in the elderly population, with an incidence that is escalating significantly alongside the global trend of population aging (1). In 2021, the global age-standardized incidence of hip fractures was estimated at approximately 948 per 100,000 individuals (2). Femoral neck fractures account for 50%–60% of these cases, with valgus-impacted fractures, typically classified as Garden stage I, representing 5%–15% of all femoral neck injuries (3, 4). Current clinical practice guidelines recommend internal fixation as the first-line treatment for these stable, undisplaced, or valgus-impacted fractures, regardless of chronological age or bone mineral density, owing to its minimal invasiveness and low rate of secondary displacement compared to primary arthroplasty (5).

Managing valgus-impacted fractures in osteoporotic elderly patients remains clinically challenging. Despite their conventional “stable” classification, these fractures frequently exhibit complex three-dimensional malalignments, particularly excessive valgus and posterior tilt. These deformities induce femoral neck shortening and alter local hip biomechanics (6, 7). Uncorrected displacement elevates risks of avascular necrosis (8), and early-onset osteoarthritis (9), leading to gait abnormalities and impaired mobility (7, 9, 10), which severely compromises long-term quality of life. The necessity of reduction for relatively stable fractures remains controversial. Current evidence suggests that *in-situ* internal fixation is viable for mildly impacted, radiographically stable fractures (valgus ≤ 15°, minimal posterior tilt), despite risks of femoral neck shortening and functional limitations (11, 12). Conversely, for fractures with valgus > 15°, unlocking and anatomical restoration significantly benefit the preservation of femoral neck length (13, 14). However, due to the “locked” and “impacted” nature of these fractures, conventional closed reduction maneuvers often fail to achieve anatomical realignment (15).

Following successful manual reduction, freehand screw insertion under C-arm guidance frequently requires repetitive guide-pin adjustments. Such drilling causes cumulative bone loss and elevates risks of fixation failure, non-union, and secondary osteoarthritis (16). Recently, orthopedic robots have revolutionized minimally invasive surgery by enhancing screw placement accuracy while significantly reducing intraoperative fluoroscopy and adjustments. By eliminating repetitive drilling, these systems preserve trabecular bone integrity, optimizing the mechanical environment for stable fixation in osteoporotic patients (17–20).

To address these challenges, we integrated manual reverse reduction with robotic-assisted fixation for elderly valgus-impacted fractures. This retrospective study evaluates the clinical and radiological efficacy of this approach versus conventional *in-situ* freehand fixation. We hypothesized that this integrated strategy would enhance anatomical restoration and screw placement precision, thereby potentially reducing complications such as non-union, fixation failure, and avascular necrosis, while improving long-term functional outcomes.

## Materials and methods

### Study design

This retrospective cohort study was approved by the Medical Ethics Committee of Tianjin Hospital (Ethics Number: 2021 Medical Ethics Review 106). Written informed consent was obtained from all patients prior to their surgical procedures. We reviewed the clinical records of patients who underwent surgical intervention for femoral neck fractures at our institution between April 2017 and December 2020.

The inclusion criteria were: (1) bone mineral density (BMD): T-score of the contralateral hip ≤ −2.5 or the presence of a clinical fragility fracture, regardless of the exact T-score; (2) fracture type: acute, closed valgus-impacted femoral neck fracture, confirmed by preoperative anteroposterior/lateral radiographs and three-dimensional computed tomography (CT) reconstruction; (3) age: between 60 and 75 years.

The exclusion criteria were: (1) pathological conditions: pathological, old, or stress fractures; secondary osteoporosis; (2) joint history: pre-existing hip dysplasia, trauma, or surgical history on either the affected or contralateral side; (3)general health: Severe multi-organ trauma or cerebrovascular diseases resulting in significant functional limitation of the affected limb; (4) data integrity: incomplete follow-up records or a follow-up period of less than 24 months.

The choice of internal fixation over total or hemi-arthroplasty in this elderly, osteoporotic cohort was strictly based on specific morphological and clinical indications. Inclusion was limited to patients presenting with stable, valgus-impacted femoral neck fractures (Garden stage I or II), where the inherent bony contact provided a biological baseline for joint preservation. Furthermore, the decision to perform internal fixation was augmented by the availability of the TiRobot navigation system, which overcomes the limitations of osteoporotic bone by ensuring optimized, parallel screw configuration and precise spatial trajectory planning. Arthroplasty was reserved only for patients with displaced, unstable fractures (Garden stage III or IV) or advanced pre-existing hip osteoarthritis.

Ultimately, 72 patients were included in this study, consisting of 36 patients in the Reduction-Assisted (RA) group (manual reverse reduction plus robot-assisted fixation) and 36 historical case-matched controls in the *in-situ* Freehand (IF) group (traditional freehand fixation without reduction). To minimize selection bias inherent to the retrospective design, patients in the IF group were selected via a strict 1:1 individual case-matching protocol based on the clinical records of the same period. The sequential matching criteria were strictly ordered as follows: (1) fracture classification (strictly Garden stage I valgus-impacted fractures); (2) sex (exact match); (3) age (tolerance within ± 3 years); and (4) interval from admission to surgery (tolerance within 2 days). To minimize performance bias, a single senior orthopedic team, experienced in both conventional and robotic techniques, performed all surgeries.

### Reduction technique

**Figure 1:**
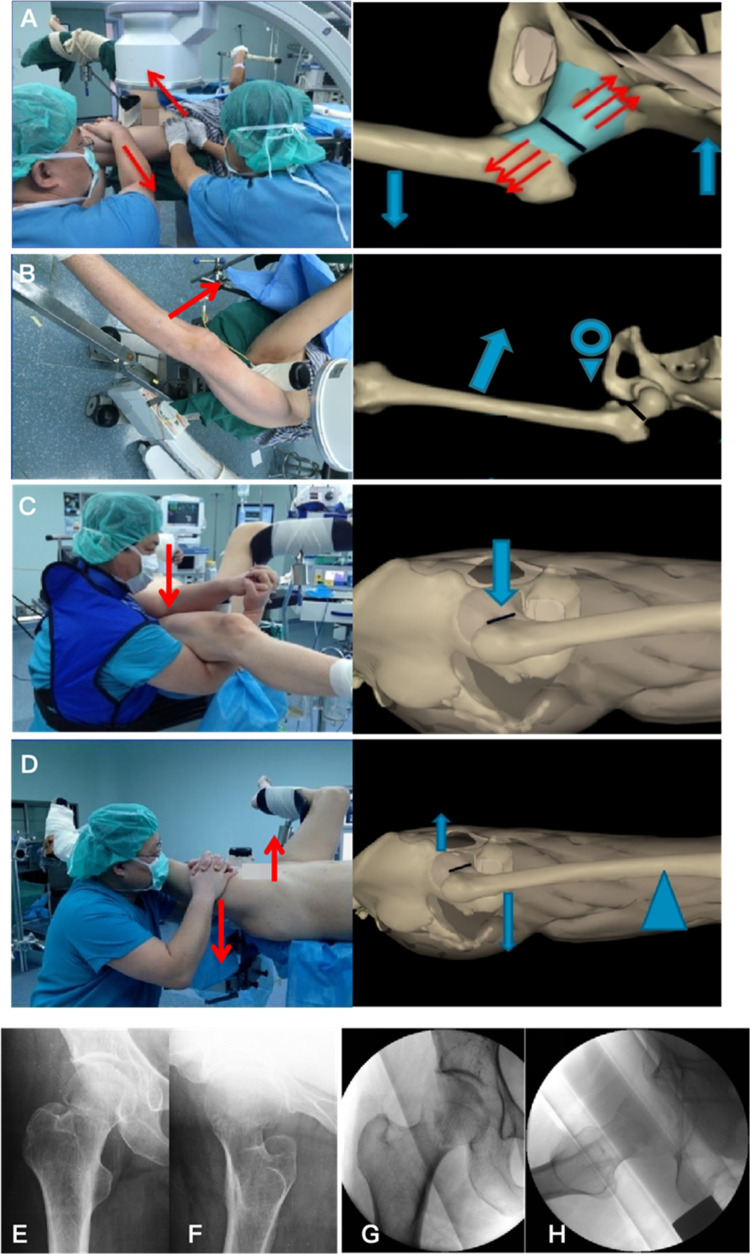
Manual reverse closed reduction maneuvers in the RA group. **(A)** Manual longitudinal traction combined with a specific “push-pull” force is applied to unlock the impacted fracture fragments and initiate the correction of the valgus angle. **(B)** Utilizing the perineal post as a stable fulcrum, the affected limb is placed in extreme adduction. **(C)** Direct downward pressure is applied to the anterior aspect of the femoral neck to correct the posterior tilt deformity. **(D)** Utilizing the surgeon's shoulder as a stable fulcrum, downward pressure is applied to the anterior femoral neck through a lever principle to further restore the anatomical sagittal profile. **(E,F)** AP and lateral x-rays **(G,H)** Intraoperative C-arm fluoroscopy images demonstrating successful dis-impaction and restoration of anatomical alignment.

Following the induction of general or epidural anesthesia, the patient was positioned supine on an orthopedic traction table with the affected limb isolated and stabilized. Preoperative C-arm fluoroscopy was performed to assess the degree of valgus displacement and posterior tilt at the fracture site. For the RA group as illustrated in Figure 1, a systematic manual reduction was executed under real-time fluoroscopic guidance. Specifically, appropriate longitudinal traction was applied, followed by the “reverse pushing method” and extreme adduction of the limb to correct the valgus deformity. Subsequently, the “anterior squeezing method” and “shoulder-top” manual pressure were applied to the proximal fragment to rectify the posterior tilt. Once multi-planar fluoroscopy confirmed satisfactory anatomical reduction, the limb position was strictly maintained, and a 2.0-mm Kirschner wire was percutaneously drilled across the fracture site to provide temporary fixation and prevent loss of reduction. In contrast, for the IF group, the affected limb was secured on the traction table without any manual manipulation, and the fracture was stabilized in its original valgus-impacted position as confirmed by C-arm fluoroscopy prior to internal fixation.

### Robot-assisted and freehand internal fixation

**Figure 2:**
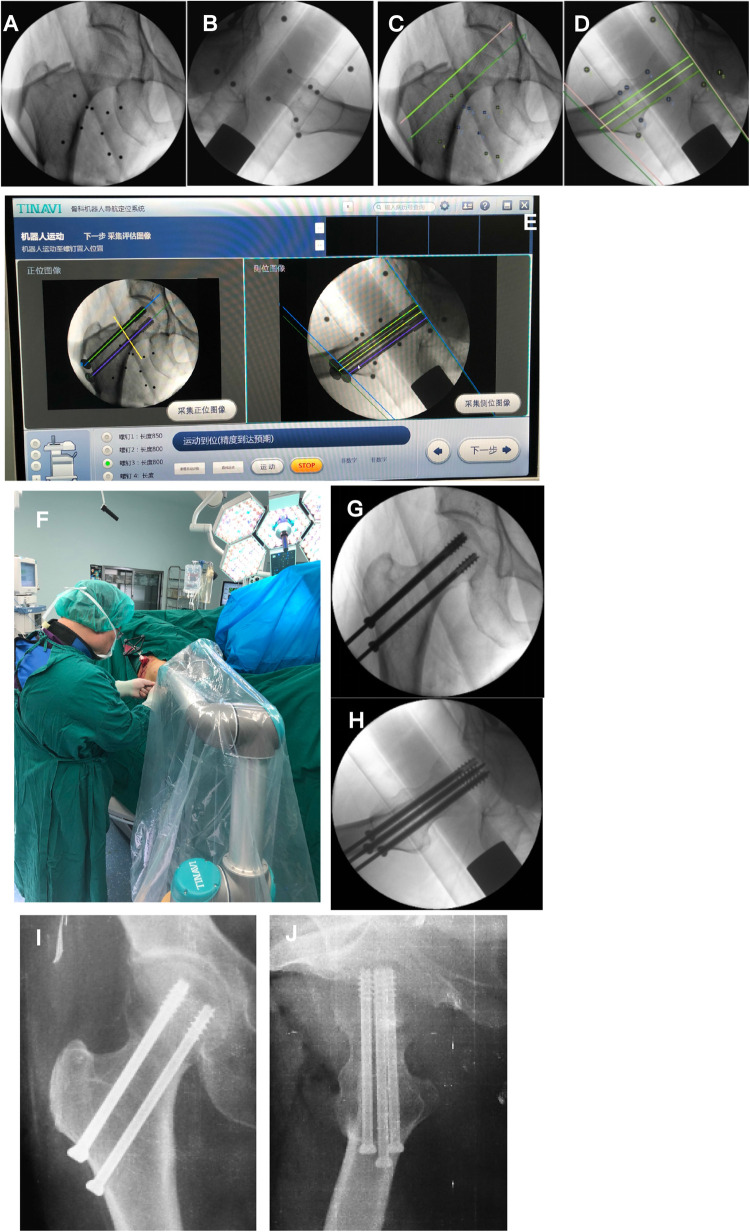
Robotic-assisted navigation workflow and internal fixation precision in the RA group. **(A,B)** Intraoperative C-arm fluoroscopy images (AP and lateral views) are captured with a specialized calibration marker to establish the robotic coordinate system. **(C,D)** The surgical workstation identifies the anatomical landmarks and defines the optimal entry points and trajectories for the three cannulated screws in an inverted triangle manner. **(E,F)** The integrated planning screen displays the synchronized multi-planar views used to guide the robotic arm to moves to the pre-planned trajectory, providing a stable physical guide for the insertion of guide pins. **(G,H)** Fluoroscopic confirmation of the guide pins following the robotic defined paths. **(I,J)** Two-day postoperative AP and lateral radiographs demonstrating the final screw configuration.

The TINAVI orthopedic robotic system (Beijing Tianzhihang Technology Co., Ltd., China) was utilized to facilitate precise screw placement in the RA group as illustrated in Figure 2. Following the manufacturer's protocol, a robotic tracer was securely fixed to the ipsilateral anterior superior iliac spine. Standard anteroposterior and lateral fluoroscopic images of the affected hip were acquired using the C-arm and transmitted to the robotic workstation for image-to-patient registration. Within the planning software, a stabilized inverted triangle configuration was designed; the software automatically calculated the optimal entry points, three-dimensional trajectories, and screw lengths. Guided by the robotic arm, the sleeve was precisely positioned, allowing for the percutaneous insertion of three parallel guide pins. The orientation and depth of each pin were verified via real-time fluoroscopy to ensure adherence to the preoperative plan. Following measurement with a depth gauge, three partially threaded cannulated screws (7.3 mm, DePuy Synthes, USA) were advanced along the pins to achieve rigid internal fixation. Throughout the procedure, strict immobility of the patient's pelvis and an unobstructed line-of-sight for the optical tracer were maintained to ensure navigational accuracy. AP/lateral x-rays were performed to evaluate the screw placement on the second day postoperatively.

In contrast, the IF group underwent conventional cannulated screw fixation under C-arm guidance. Three guide pins were inserted in an inverted triangle pattern using a freehand technique followed by three partially threaded cannulated screws (7.3 mm, DePuy Synthes, USA) were advanced. This process frequently required repetitive repositioning and multiple fluoroscopic verifications until a satisfactory screw trajectory and distribution were achieved.

### Postoperative management and rehabilitation

Patients received standardized care, including analgesia, anti-edema therapy, and thromboprophylaxis initiated 24 hours postoperatively. Isometric quadriceps exercises began after anesthesia recovery. Assisted mobilization (e.g., bedside sitting) commenced on postoperative day 2 within pain-free limits; however, leg crossing, side-lying, and early straight-leg raises were restricted. Weight-bearing was progressed based on radiological healing, typically transitioning from non-weight-bearing to partial weight-bearing at 6 weeks and full weight-bearing after 12 weeks. Comprehensive anti-osteoporosis therapy was maintained throughout, including regular BMD monitoring, intravenous bisphosphonates, and oral calcium/vitamin D supplementation.

### Clinical and radiological assessment

Patient demographics, including age, sex, and the interval from admission to surgery, were recorded. Surgical details, such as operative time, estimated blood loss, total fluoroscopy shots, and the number of guide-pin adjustments, were documented for both groups. Clinical functional outcomes were assessed using the Harris Hip Score (HHS) at 12 and 24 months postoperatively to evaluate pain, function, deformity, and range of motion. Based on the cumulative score, clinical outcomes were categorized according to the following grading system: Excellent: 90–100 points; Good: 80–89 points; Fair: 70–79 points; Poor: < 70 points (21).

Postoperative radiological evaluation was performed on the second day following surgery using AP/lateral x-rays and 3D-CT scans to analyze the quality of anatomical reduction and screw placement precision. Scheduled follow-up assessments occurred at 6 weeks, 12 weeks, 6 months, 1 year, and 2 years postoperatively. During these visits, routine AP and lateral radiographs were obtained to monitor fracture healing, potential femoral neck shortening, internal fixation stability, and signs of femoral head necrosis or post-traumatic osteoarthritis. Fracture healing was formally defined as the complete disappearance of the fracture line as confirmed by both x-ray and CT imaging. Conversely, non-union was diagnosed if healing was not achieved at least 9 months postoperatively, or if no progressive signs of healing were observed over three consecutive months of dynamic observation. In all suspected cases of non-union or femoral head necrosis, diagnosis was strictly confirmed through advanced imaging, including CT and magnetic resonance imaging (MRI), to identify structural changes or signal abnormalities.

Valgus and posterior tilt angles were measured pre- and postoperatively based on methods by Buord et al. (22) and Palm et al. (23). The valgus angle was defined by the intersection of tangent lines from the fracture surfaces on AP radiographs. The posterior tilt was determined on lateral radiographs by measuring the angle between the mid-femoral neck centerline and the radius centerline of the femoral head.

**Figure 3:**
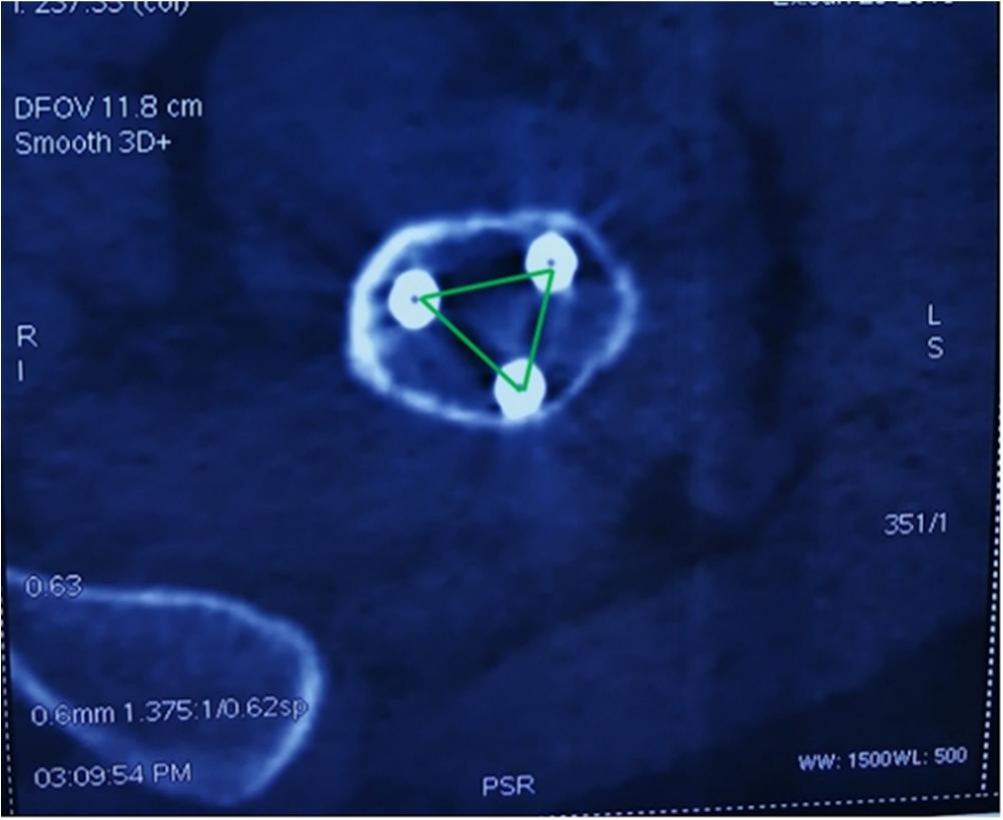
Evaluation of screw spatial distribution. Axial CT scan at the narrowest portion of the femoral neck demonstrates the calculation of the screw dispersion index (SDI). The green triangle represents the area formed by the centers of the three cannulated screws.

Screw placement precision was evaluated on day 2 radiographs by measuring the angle between each screw and the femoral neck axis; the total angular deviation was defined as the sum of these three angles, which measured the angular error between the planned and executed screw trajectories. Minimizing this deviation guarantees the execution of planned parallelism, ensuring uniform stress distribution and preventing localized stress concentration or screw cut-out. Screw dispersion index (SDI) was analyzed via postoperative 3D-CT, as shown in Figure 3, a triangle was formed by connecting the centers of the three screws at the narrowest cross-section of the femoral neck. The ratio of the triangle's area to the total neck cross-sectional area was defined as the screw dispersion, representing the spatial distribution efficiency. Maximizing the SDI expands the effective dynamic fixation area within the femoral neck, thereby enhancing rotational stability and shearing resistance under early weight-bearing conditions. Any cortical perforation was documented via 3D-CT reconstruction.

The degree of femoral neck shortening (FNS) was evaluated at the final follow-up using the femoral head displacement method (24). Standardized AP radiographs of both hips were superimposed via PACS, aligning pelvic anatomical landmarks. FNS was calculated as the vector sum of the horizontal and vertical displacement of the femoral head center on the affected side relative to the contralateral side. This approach provided precise quantification of the anatomical loss of the femoral neck during healing. FNS is a critical radiographic indicator directly reflecting loss of abductor muscle tension. Severe FNS (> 5 mm) leads to a decreased lever arm for the gluteus medius and minimus, resulting in persistent hip pain, abductor weakness, and a trend of limp during ambulation.

#### Statistical analysis

Statistical analysis was performed using STATA v12.0 (StataCorp, USA). Continuous variables were expressed as mean ± standard deviation; normality was assessed via the Shapiro–Wilk test. Intergroup comparisons utilized independent-sample t-tests for normally distributed data and Wilcoxon rank-sum tests otherwise. Categorical variables (e.g., sex, complications, excellent/good rates) were compared using Pearson chi-square tests. Significance was set at a two-tailed *α* = 0.05.

Inter- and intra-observer reliability for radiological parameters (screw angles, SDI, valgus/posterior tilt, and FNS) was evaluated using the Intraclass correlation coefficient (ICC). Two observers independently performed assessments twice with a 3-month interval. All ICC values exceeded 0.75, confirming excellent measurement consistency; the final analysis used the mean of these four measurements. Furthermore, due to the modest sample size (*n* = 72), multivariable regression analysis was not performed to evaluate independent predictors. However, the excellent homogeneity of baseline characteristics between the two groups significantly mitigates the potential influence of major preoperative confounders.

## Results

### Patient demographics and baseline characteristics

Initially, 53 elderly patients with osteoporotic, valgus-impacted femoral neck fractures treated via the RA technique between April 2017 and December 2020 were screened. Based on the selection criteria, 36 patients were enrolled in the RA group. Thirty-six case-matched patients treated with *in-situ* freehand fixation during the same period served as the control (IF) group. As shown in Table 1, no significant intergroup differences existed regarding preoperative baseline characteristics, including age, sex, BMI, and initial deformities (*p* > 0.05).

**Table 1:** Baseline demographic and preoperative clinical characteristics of patients in the RA and IF groups (*n* = 36 each).

Characteristics	RA group	IF group	Value	*p*
Age, y	62.92 ± 6.19	61.75 ± 3.77	0.3444^a^	0.7306
Sex			0.0566^b^	0.8119
Male n(%)	16 (44.44)	15 (41.67)		
Female n(%)	20 (55.56)	21 (58.33)		
Valgus angle (°)	12.21 ± 6.57	12.91 ± 6.34	−0.4600^c^	0.6469
Posterior tilt angle (°)	17.58 ± 11.97	17.11 ± 10.93	0.1740^c^	0.8625
BMD T scores	−2.76 ± 0.28	−2.83 ± 0.34	1.5192^a^	0.1287
Time from injury to surgery, d	3.06 ± 0.67	2.97 ± 0.65	0.5303^a^	0.5959

Data are expressed as mean ± SD or n; RA: reduction-assisted; IF: *in-situ* freehand; BMD: bone mineral density;.

a indicates Wilcoxon rank-sum test.

b indicates Pearson chi-square test.

c indicates independent-sample *t*-test.

### Technical precision and safety

Robotic navigation demonstrated greater technical accuracy compared with the freehand approach (Table 2). Although the RA group required a longer operation duration (1.74 ± 0.63 hours vs 1.29 ± 0.37 hours, *p* = 0.0014), it provided significantly higher placement precision. The total angular deviation from the ideal femoral neck axis was markedly reduced in the RA group compared to the IF group (5.92 ± 3.75° vs 11.14 ± 5.28°, *p* < 0.0001), a result achieved with substantially fewer guide-pin adjustments (0.17 ± 0.38 vs 1.89 ± 1.09, *p* < 0.0001). Furthermore, the robotic system optimized the mechanical stability of the construct, as evidenced by a significantly higher SDI (22.10 ± 1.47 vs. 18.83 ± 1.22, *p* < 0.0001), indicating a more effective spatial distribution of the screws within the femoral neck.

**Table 2:** Comparison of intraoperative and radiographic parameters between the RA and IF groups (*n* = 36 per group).

Parameters	RA group	IF group	Value	*p*
Operation duration, h	1.74 ± 0.63	1.29 ± 0.37	3.1867^a^	0.0014
Blood loss, mL	150.00 ± 49.28	133.33 ± 43.75	1.5278^a^	0.1266
Number of fluoroscopy shots, n	33.28 ± 7.71	56.56 ± 19.71	−5.4047^a^	<0.0001
Number of guide-pin adjustments, n	0.17 ± 0.38	1.89 ± 1.09	−7.0775^a^	<0.0001
Total angular deviation,°	5.92 ± 3.75	11.14 ± 5.28	−4.3251^a^	<0.0001
SDI, %	22.10 ± 1.47	18.83 ± 1.22	10.2706^a^	<0.0001
Cortical perforation			5.2578^b^	0.0218
Yes, n(%)	2 (5.56)	9 (25.00)		
No, n(%)	34 (94.44)	27 (75.00)		

Data are expressed as mean ± SD or n. RA, reduction-assisted; IF, *in-situ* freehand; SDI, screw dispersion index.

a indicates Wilcoxon rank-sum test.

b indicates Pearson chi-square test.

Regarding safety, robotic guidance significantly reduced intraoperative fluoroscopy shots (33.28 ± 7.71 vs 56.56 ± 19.71, *p* < 0.0001), lowering radiation exposure for patients and surgical staff. Additionally, the incidence of cortical perforation was significantly lower in the RA group (5.56% vs 25.00%, *p* = 0.0218), demonstrating enhanced cortical safety via high-precision trajectory planning. Furthermore, all 36 patients initially screened and allocated to the RA group successfully completed the planned robot-assisted procedure. There were no documented instances of navigational planning failure, reference frame displacement, or intraoperative conversion to the conventional freehand technique.

### Radiographic quality of anatomical reduction

Manual reverse closed reduction achieved favorable anatomical realignment for valgus-impacted deformities. Within the RA group, significant postoperative improvements were observed in both coronal and sagittal alignment (Table 3). The mean valgus angle decreased from 12.21 ± 6.57° preoperatively to 3.52 ± 3.34° postoperatively (*p* < 0.001), while the mean posterior tilt angle was corrected from 17.58 ± 11.97° to 2.96 ± 0.52° (*p* < 0.001).

**Table 3:** Comparison of preoperative and two-day postoperative radiographic deformities within the RA group (*n* = 36).

Parameters	Pre-op	Post-op	t	*p*
Valgus angle (°)	12.21 ± 6.57	3.52 ± 3.34	7.0744	<0.0001
Posterior tilt angle (°)	17.58 ± 11.97	2.96 ± 0.52	7.3214	<0.0001

Data are expressed as mean ± SD. RA, reduction-assisted.

Further inter-group analysis highlighted a substantial disparity in reduction quality between the two groups (Table 4). Postoperative evaluations showed that the RA group achieved significantly better alignment than the IF group. Specifically, the postoperative valgus angle in the RA group was markedly lower than that in the IF group (3.52 ± 3.34° vs 12.91 ± 6.34°, *p* < 0.001). Similarly, the RA group demonstrated a significantly smaller postoperative posterior tilt angle compared to the IF group (2.96 ± 0.52° vs 17.11 ± 10.93°, *p* < 0.001).

**Table 4:** Comparison of two-day postoperative valgus and posterior tilt angles between the RA and IF groups (*n* = 36 per group).

Parameters	RA group	IF group	t	*p*
Valgus angle (°)	3.52 ± 3.34	12.91 ± 6.34	−7.8622	<0.0001
Posterior tilt angle (°)	2.96 ± 0.52	17.11 ± 10.93	−7.7588	<0.0001

Data are expressed as mean ± SD. RA, reduction-assisted group; IF, *in-situ* freehand group.

### Clinical functional recovery and complications

The clinical functional assessment at 24 months postoperatively demonstrated a favorable recovery trend in the RA group compared to the IF group based on the Harris Hip Score evaluation (Table 5). The mean HHS was significantly higher in the RA group (82.00 ± 11.44) than in the IF group (73.61 ± 15.07, *p* = 0.0097). Furthermore, the RA group achieved a significantly higher excellent/good functional rate of 75.00% (27/36), whereas the IF group reached only 44.44% (16/36) (*p* = 0.0082).

**Table 5:** Comparison of Harris hip scores and clinical outcomes between the RA and IF groups at 24 months postoperatively (*n* = 36 per group).

Outcomes	RA group	IF group	Value	*p*
HHS	82.00 ± 11.44	73.61 ± 15.07	2.6606^a^	0.0097
Excellent/good functional rates			6.9864^b^	0.0082
Yes, n(%)	27 (75.00)	16 (44.44)		
No, n(%)	9 (25.00)	20 (55.56)		

Data are expressed as mean ± SD or n. RA, reduction-assisted; IF, *in-situ* freehand; HHS, Harris hip scores.

a indicates independent-sample *t*-test.

b indicates Pearson chi-square test.

As for long-term outcomes and complications, the RA group demonstrated significant advantages in both structural stability and joint preservation compared to the IF group. As detailed in Table 6, the overall complication rate was substantially lower in the RA group (19.44% vs.47.22%, *p* = 0.0124). Specifically, the RA group exhibited significantly lower incidences of femoral head necrosis (5.56% vs 25.00%, *p* = 0.0218), non-union (8.33% vs 27.78%, *p* = 0.0320), and internal fixation failure (5.56% vs 22.22%, *p* = 0.0409).

**Table 6:** Comparison of postoperative complications between the RA and IF groups at the final follow-up (*n* = 36 per group).

Complications/Parameters	RA group	IF group	Value	*p*
Complications			6.2500^a^	0.0124
Yes, n(%)	7 (19.44)	17（47.22）		
No, n(%)	29 (80.56)	19（52.78）		
Femoral head necrosis			5.2578^a^	0.0218
Yes, n(%)	2 (5.56)	9 (25.00)		
No, n(%)	34 (94.44)	27 (75.00)		
Non-union			4.5997^a^	0.0320
Yes, n(%)	3 (8.33)	10 (27.78)		
No, n(%)	33 (91.67)	26 (72.22)		
Internal fixation failure			4.1806^a^	0.0409
Yes, n(%)	2 (5.56)	8 (22.22)		
No, n(%)	34 (94.44)	28 (77.78)		
FNS, mm	2.5 ± 2.1	9.1 ± 3.8	−9.1209^b^	<0.0001

Data are expressed as mean ± SD. RA, reduction-assisted group; IF, *in-situ* freehand group; FNS, femoral neck shortening.

a indicates Pearson chi-square test.

b indicates independent-sample *t*-test.

**Figure 4:**
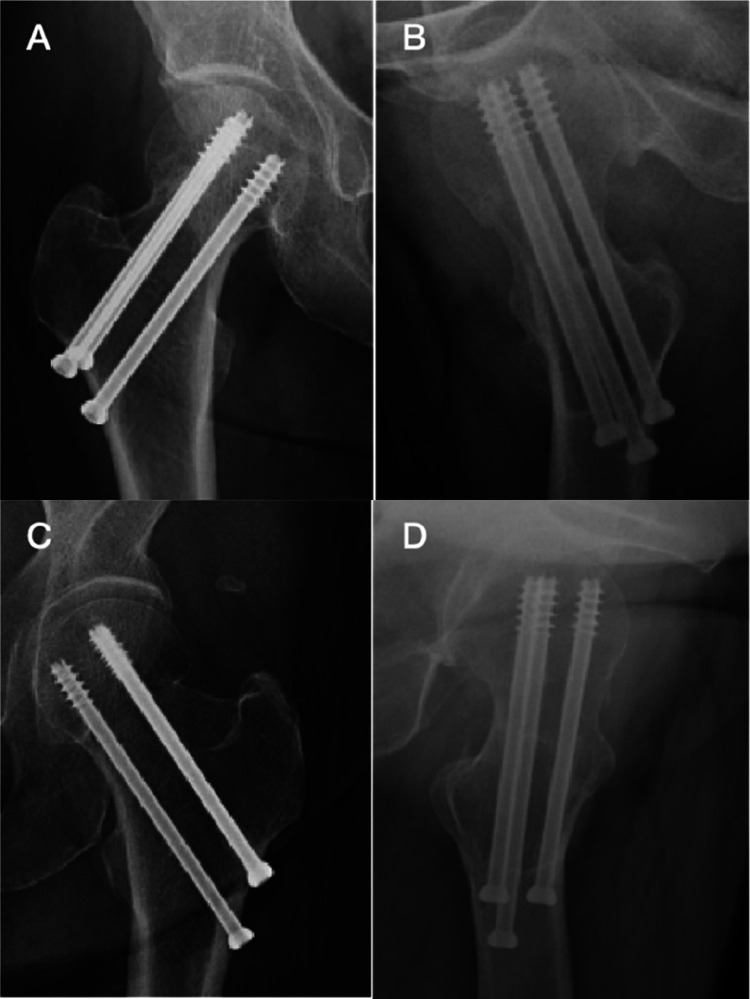
Representative radiographic comparison at the 24-month follow-up. **(A,B)** RA Group: Patient 1, female, 63 years old, AP and lateral radiographs show excellent anatomical reduction, stable fracture union, and minimal femoral neck shortening. **(C,D)** IF Group: Patient 2, female, 60 years old, AP and lateral radiographs also show good fracture union but persistent deformities and femoral neck shortening.

Furthermore, the degree of FNS was markedly reduced in the RA group (2.5 ± 2.1 mm) relative to the IF group (9.1 ± 3.8 mm, *p* < 0.0001), reflecting superior maintenance of the anatomical structure. Among patients who developed complications, only 6 individuals in the RA group eventually required conversion to total hip arthroplasty, compared to 15 patients in the IF group. Crucially, regarding systemic safety profiles during the immediate postoperative hospitalization period, there were no documented cases of acute myocardial infarction, cerebrovascular accidents, symptomatic pulmonary embolism (PE), or deep vein thrombosis (DVT) in either group. These clinical findings are corroborated by the two-year postoperative radiographs. As illustrated in Figure 4, the RA group demonstrated excellent fracture union and minimal shortening compared to the IF group.

## Discussion

This study validates a “dual-optimization” framework—integrating precise manual anatomical reduction with robotic-assisted screw placement—specifically for elderly patients with osteoporotic valgus-impacted femoral neck fractures. Our results suggest that this integrated strategy can favorably assist in correcting initial deformities and optimizing screw spatial distribution, thereby supporting structural stability. Furthermore, it was associated with a lower incidence of major complications, including avascular necrosis, non-union, and severe femoral neck shortening in our cohort. These findings provide valuable radiographic and clinical insights indicating that proactive anatomical restoration and technical precision are beneficial for optimizing recovery in this patient population.

Valgus-impacted femoral neck fractures are characterized by valgus angulation and posterior tilt of the femoral head. Based on Raaymakers’ classification (25), these injuries are divided into three radiographic types: Type 1 (valgus and posterior tilt), Type 2 (valgus only), and Type 3 (posterior tilt only). Evidence suggests that over 90% of cases are Type 1 or 2, where valgus >15° (10, 26) or posterior tilt >13° (27, 28), represent critical thresholds for internal fixation failure, avascular necrosis, and reoperation. These risks are exacerbated in elderly osteoporotic patients (10, 29, 30), necessitating anatomical reduction prior to fixation to prevent poor outcomes.

Concerns persist that manual reduction might compromise femoral head blood supply. However, recent evidence suggests that anatomical reduction significantly reduces fracture-site stress and facilitates Weitbrecht capsular vascular network reconstruction, potentially lowering rates of non-union and avascular necrosis (14, 31). Minimally invasive techniques, such as bone hook traction, have shown no increase in necrosis while significantly enhancing functional scores (6, 13, 32, 33). Nevertheless, reduction remains technically demanding; “negative buttress” following repeated unsuccessful attempts exacerbates risks of structural collapse and necrosis, particularly in elderly osteoporotic patients (8, 13, 34, 35). The manual closed reverse reduction method proposed in this study offers a more minimally invasive and simplified alternative to traditional “joystick” techniques involving Kirschner wires. By maintaining moderate traction on a fracture table and utilizing the “fulcrum-lever” principle, the surgeon reverses the specific valgus and retroversion displacement based on preoperative imaging. This approach avoids additional trauma from percutaneous tools while effectively restoring the anatomical profile. In our cohort, the RA group achieved significant improvements in both valgus and posterior tilt angles post-reduction. This high-quality restoration directly translated into superior long-term results, including higher HHS and excellent/good rates, as well as significantly lower rates of femoral neck shortening, necrosis, and non-union compared to the IF group. These outcomes provide supportive evidence for the efficacy and safety of the manual closed reverse reduction technique.

The second pillar of our “dual-optimization” strategy is leveraging robotic navigation to ensure technical precision in internal fixation. A primary challenge in osteoporotic femoral neck fractures is the sparse trabeculae and diminished bone mass centrally. Consequently, the mechanical stability of cannulated screws relies primarily on “bi-cortical” anchorage: the screw head within the subchondral bone and the tail in the lateral femoral cortex (36, 37). Repetitive intraoperative guide-pin adjustments inevitably cause secondary cortical damage, significantly compromising anchoring strength and construct stability. Our results suggest that robotic assistance may help mitigate this risk by reducing unnecessary intraoperative adjustments, thereby contributing to stable fixation even in poor-quality bone.

Structural stability in femoral neck fractures depends heavily on screw spatial configuration, typically arranged in an inverted triangle. To maximize biomechanical strength, ideal positioning requires screws to remain parallel to the femoral neck axis, with an angular deviation <10° and a distance from the neck cortex <3 mm (38, 39). Achieving such precision freehand is inherently challenging, particularly in complex fractures. Recent studies demonstrate that robotic navigation significantly enhances both screw parallelism and dispersion compared to freehand placement (18, 19, 40, 41), which is aligned with our findings as evidenced by the RA group, guided by the TiRobot system, achieved a marked improvement in the total angular deviation and a higher screw dispersion. This mechanical advantage, characterized by high parallelism and maximum dispersion within the cortical boundaries, coupled with the anatomical restoration achieved through our reduction technique, explains why the RA group suffered significantly less **FNS** and fewer instances of fixation failure compared to the IF group.

Beyond precision, robotic navigation provides distinct safety and perioperative benefits. In this study, the RA group experienced significantly less blood loss and required fewer fluoroscopy shots than the IF group. Furthermore, literature indicates that robotic assistance correlates with shorter hospital stays, accelerated recovery, reduced early postoperative pain, and earlier weight-bearing (19, 42, 43). These systemic benefits highlight the holistic advantage of integrating technology into geriatric fracture care. However, robotic implementation faces challenges, including a steep learning curve, high capital costs, and specialized institutional requirements (44, 45). These factors contributed to the longer operative time in our RA group, which typically diminishes with increasing surgical proficiency and technological evolution. Notably, while these 36 RA cases were performed in a strictly sequential manner, we did not observe a statistically significant or uniform chronological decline in operative time across the consecutive cases. The trade-off between increased operative time and the reduction in long-term complications, such as lower THA conversion rates, suggests that this robotic-assisted pathway may offer a meaningful clinical advantage for optimizing elderly patient outcomes.

Despite these promising outcomes, several limitations warrant acknowledgment. Firstly, the retrospective design introduces inherent selection bias, although baseline characteristics were comparable, a prospective randomized controlled trial (RCT) is necessary to establish a higher level of evidence. Secondly, this single-center study had a relatively small sample size (*n* = 72), potentially limiting the generalizability of our findings. Large-scale, multi-center studies are required to confirm these functional and radiographic benefits across diverse populations. Thirdly, as a major methodological limitation, the RA group utilized a combined strategy of manual reverse closed reduction and robotic-assisted fixation, whereas the IF group underwent neither. Consequently, the independent contributions of the reduction maneuver versus the precision of robotic spatial guidance cannot be completely isolated based on our current data. While this combined approach reflects real-world clinical optimization for achieving optimal biomechanical alignment, future comparative trials are warranted to delineate the independent effects of each component. Finally, while the 24-month minimum follow-up was sufficient for detecting non-union and early necrosis, it might miss late-onset femoral head collapse or post-traumatic arthritis. Longer-term monitoring (e.g., 5–10 years) would provide a more comprehensive understanding of this method's joint preservation potential.

## Conclusion

Our clinical data indicate that this integrated strategy, combining manual closed reverse reduction with robotic-assisted screw placement, can favorably enhance fixation stability and precision while alleviating the risk of major complications, therefore representing a beneficial option for joint preservation in this geriatric cohort.

## Data Availability

The original contributions presented in the study are included in the article/Supplementary Material, further inquiries can be directed to the corresponding authors.
